# The relationship between career calling and presenteeism: the role of workaholism and self-compassion

**DOI:** 10.1080/00049530.2024.2445247

**Published:** 2025-01-19

**Authors:** Zhaobiao Zong, Feifei Sun, Haichao Sun, Shaoqing Su, Baojian Wei

**Affiliations:** a School of Psychology and Cognitive Science, East China Normal University, Shanghai, P. R. China; b Information Center, Affiliated Hospital of Shandong University of Traditional Chinese Medicine, Jinan, P. R. China; c School of Teacher Education, Taishan University, Taian, P. R. China; d School of Educational Science, Northwest Normal University, Lanzhou, P. R. China; e College of Educational Sciences, Northwest Normal University, Lanzhou, P. R. China; f School of Nursing, Shandong First Medical University & Shandong Academy of Medical Sciences, Taian, P. R. China

**Keywords:** Nursing, career calling, presenteeism, workaholism, self-compassion

## Abstract

**Objective:**

While the relationship between career calling and its impact on organisations and employees has been well-documented, the connection between career calling and presenteeism remains unclear. This study aims to elucidate the positive relationship between career calling and presenteeism, as well as explore potential mitigation strategies. By drawing on work as calling theory and self-compassion literature, we investigate the mediating role of workaholism and the moderating effect of self-compassion in the relationship between career calling and presenteeism.

**Methods:**

A time-lagged cross-sectional questionnaire was administered in three waves, with two-week intervals between each wave. A total of 218 valid responses were collected from nurses working in three hospitals located in northern China. Initially, confirmatory factor analysis was performed to establish discriminant validity. Subsequently, the SPSS macro Process 3.0 was utilised to test the mediating hypothesis, employing 5,000 bootstrap iterations to obtain 95% bias-corrected confidence intervals. Simple slope analysis was conducted to evaluate the moderating hypothesis. Finally, the estimated indirect effect and moderated mediation coefficients were calculated at both high and low values of the moderating variable to assess the moderated mediation hypothesis.

**Results:**

Our research reveals the underlying mechanism of workaholism and the mitigative effects of self-compassion in the career calling and presenteeism linkage. The results indicate a positive indirect relationship between career calling and presenteeism via workaholism, and that this indirect effect is weaker when individuals exhibit higher levels of self-compassion.

**Conclusion:**

The study sheds light on the relationship between career calling and workaholism and presenteeism among nurses, suggesting that self-compassion plays a pivotal role in the above relationship.

## Key Points

**What is already known about this topic:**

1. Nurses with a career calling place greater emphasis on job satisfaction derived from factors such as motivation and social responsibility. As a result, they are better able to fulfil their work purpose and express their true selves.
2. Presenteeism not only contributes to inefficiency among nurses but also adversely affects their physical and mental health. While nurses frequently possess a strong sense of career calling, the influence of this calling on their presenteeism behaviour remains largely unexplored.
3. Self-compassion effectively mitigates the depletion of various resources resulting from over-commitment to work, thereby enhancing work performance and fostering increased psychological resilience.

**What this topic adds:**

1. When nurses experience a profound sense of career calling, they are more inclined to remain in their positions, even in the face of illness or personal challenges.
2. Career callings can prompt nurses to develop tendencies towards workaholism, which may subsequently heighten their susceptibility to presenteeism.
3. Higher levels of self-compassion can enhance nurses’ awareness of their physical and mental health, potentially moderating the positive effects of career calling on presenteeism, particularly concerning tendencies towards workaholism. As a result, nurses may attain a balance between their career calling and presenteeism.

## Introduction

We have given it our all. We have worked to the brink of exhaustion. And once again, we have understood – maybe better than ever – why we chose this profession: to care for people and to save lives.

-- Aroa López^1^

People have come to understand in recent years that pursuing one’s profession involves more than just landing a job; it also involves discovering one’s genuine passions. This quest encompasses not only monetary gain or job advancement, but also the quest of self-worth and the significance that a career provides for the individual (Duffy & Dik, 2013; Thompson & Bunderson, 2019). According to Ms. López, their quest for meaning in their work (*career calling*, an individual being motivated by both an external calling and an internal drive to practice a particular professional role with a strong sense of purpose and in a meaningful way; Dik & Duffy, 2009) is what keeps them going in a high-intensity work environment.

In addition, research has demonstrated that nurses’ career calling is fundamentally altruistic, highlighting the social value of their individual work and its benefits to patients, thereby allowing them to realise personal values and address social needs (He et al., 2024). Consequently, nurses who possess a strong sense of career calling perceive their work as a means to pursue life’s meaning, which subsequently enhances their enthusiasm and motivation (Ziedelis, 2019), improves their occupational well-being and fulfilment (Yang & Chen, 2020), and increases their propensity to proactively seek solutions to overcome challenges in unfavourable environments (Zhou et al., 2021). Simultaneously, the social expectations placed upon nurses instil in them a profound sense of mission, making them acutely aware of the trust and responsibility they bear on behalf of society. However, this sense of duty can also have detrimental effects on nursing staff, including work fatigue and organisational exploitation (Duffy et al., 2018; Zhu et al., 2021). For instance, a heightened sense of career calling is linked to increased working hours for nurses (Uzunbacak et al., 2023), which may adversely impact their working conditions as well as their physical and mental health (Lee et al., 2024). Nevertheless, many prior studies, particularly those focusing on highly career calling such as medicine and nursing, have concentrated on the specific mechanisms through which the beneficial effects of career calling manifest. However, the potential negative effects of career calling have not been explored in sufficient depth (Andel et al., 2021; Zhou et al., 2021). Because that career calling has the potential to be harmful, it is crucial to comprehend how and why the adverse effect occurs and, more importantly, how to stop it.

The Work as Calling Theory (WCT) combines the benefits of career calling at work with any possible drawbacks (Duffy et al., 2018). According to the WCT hypothesis, career calling clarifies one’s professional objectives, and career passion and recognition can improve one’s performance in the workplace. However, higher career calling might push people to to their work, negatively affecting them (Duffy et al., 2018; Ehrhardt & Ensher, 2021). Therefore, We argue that a nurse’s sense of career calling may predispose them to exert excessive effort in their work, compelling them to engage in occupational activities that may compromise their health. Moreover, during non-working hours, nurses may experience feelings of guilt and unease when distancing themselves from their professional responsibilities, which can lead to detrimental effects (workaholism, Ng et al., 2007; Schaufeli et al., 2009).

Then, nurses’ workaholism can lead to presenteeism behaviours (Johns, 2010; Wang et al., 2022). Specifically, the intrinsic psychological drive of nurses to work excessively hard often outweighs the impact of physical discomfort and other negative factors. While various studies have highlighted the benefits of presenteeism (Karanika-Murray & Biron, 2020), including enhancements in organisational citizenship behaviours and improvements in work performance (Boekhorst & Halinski, 2023; Wang et al., 2023), this paper aims to explore the negative impacts associated with presenteeism. We define presenteeism as the phenomenon where individuals physically attend work despite experiencing health issues that would typically warrant absence, resulting in reduced productivity while present. This definition encompasses both the frequency of attendance while unwell and the associated productivity losses (Lu et al., 2013). We have chosen to focus on this comprehensive view of presenteeism because it provides a holistic understanding of the impact on both individual well-being and organisational outcomes. Thus, this study generalises why career calling might result in unfavourable individual behavioural outcomes (e.g., presenteeism) via workaholism.

Furthermore, we aim to enhance our understanding of a significant topic: how to mitigate this negative linkage. In the context of overcoming distressing and traumatic experiences, individuals act as proactive agents (Schabram & Heng, 2022). Consequently, We propose that self-compassion may help individuals diminish this adverse effect. The capacity for self-compassion refers to an individual’s ability to remain receptive to their suffering and challenges, to feel kindness and concern for themselves, to approach their shortcomings and failures with an understanding, non-judgemental attitude, and to recognise that their experiences are common to all people (Lanaj et al., 2022; Neff, 2003; Schabram & Heng, 2022). Individuals with higher levels of self-compassion demonstrate an enhanced ability to recognise their own physical and mental states. They proactively motivate themselves to regulate their emotions, which effectively mitigates the negative consequences of excessive work related to their career calling (Kreemers et al., 2018).

**Figure 1.**
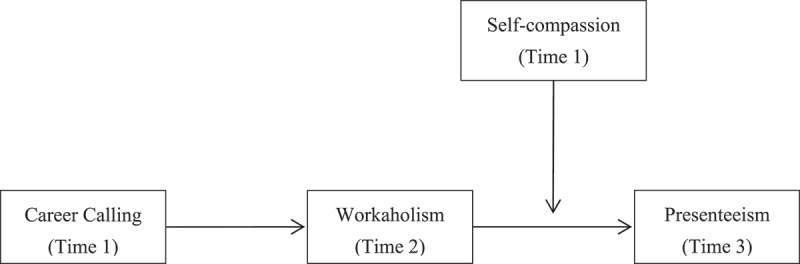
The conceptual model.

Figure 1 depicts our conceptual model in detail. Our study contributes in several ways. We started by looking at the negative effects of career calling. Exploring the effect of career calling is crucial to safeguarding their physical and mental well-being (Zhou et al., 2021). Moreover, this adds to the body of knowledge on the contradictory effects of career calling. In order to complete the puzzle of how calling might result in negative effects, we provide workaholism as a crucial link between calling and presenteeism. Finally, we offer a crucial viewpoint on deconstructing the negative consequences of calling from a self-compassion perspective.

## Theory and hypotheses development

### Work as calling theory

WCT contends that calling may improve people’s dispositions and aid their professional success (Duffy et al., 2018). People are more likely to view their jobs and careers as a source of purpose and worth when they have a strong sense of calling. People are more likely to commit to working for satisfaction when they are enthusiastic and recognised for their efforts (Duffy et al., 2018; Ehrhardt & Ensher, 2021). Those with higher career calling also have defined goals and convictions in finishing their task, a higher tolerance for risks and failures, a greater propensity for problem-solving, and superior performance at work (Beer et al., 2022; Park et al., 2016). Nevertheless, Duffy et al. (2018) pointed out that calling might occasionally have negative effects. Strong callings will always motivate a person to pursue more meaningful goals. Therefore those with strong callings will exhibit more obsessive behaviour and a propensity to overwork. To reach this objective and feel more satisfaction, the person must continue working and investing more time and energy into their task (Hirschi et al., 2019; Keller et al., 2016). While the calling motivates people to work, it also influences them to see labour as a moral obligation that cannot be shunned (Andel et al., 2021). The strain of moral obligation causes people to sacrifice personal time for lengthy hours, making it challenging to set boundaries with work and having a detrimental influence on both work and oneself (Andel et al., 2021; Clinton et al., 2017).

### Career calling and presenteeism

Those who have found their career calling view a specific line of work as meaningful in their life and strive for self-worth in their career, frequently with intense internal motivation (Humayun et al., 2022). Employees who view their jobs as callings internalise the task’s worth, and the ensuing drive is fundamentally independent (Duffy et al., 2018; Ehrhardt & Ensher, 2021). Employees with a high sense of purpose will seek their job self-fulfilment and meaning in their work, motivated by their love and appreciation of the work itself (Humayun et al., 2022). As a result, the career calling comes from within the person and is motivated by something more significant than the self (Duffy et al., 2018; Ehrhardt & Ensher, 2021). People with internal motivation are frequently motivated to pursue innovations and challenges and have sustainability linked to individual initiative and altruistic conduct. Internal drive frequently shows the excellent potential of people (Hirschi et al., 2019; Keller et al., 2016).

Presenteeism is when a person refuses to stay home from work because they are sick (Johns, 2010). Although naturally charitable and advantageous to the organisation, it may also result in inefficiencies and potentially increase the risk of medical errors. This is particularly concerning when individuals are already experiencing extreme physical and mental exhaustion yet remain reluctant to miss work (Ferreira et al., 2024). Thus, the application of presenteeism must be directed by good intentions. Career calling is characterised by its propensity for organisation and generosity (Hadjisolomou et al., 2022; Rivkin et al., 2022). Presenteeism is encouraged by employees’ propensity to prioritise the needs of the company and the group over their own interests, as well as their perception of their own responsibility and willingness to put the organisation’s needs first (Brosi & Gerpott, 2022). Duffy et al. (2011) also showed that people with a strong sense of their career calling tend to have stronger organisational commitments, particularly emotional commitments. In other words, people who see their work as a calling are more invested in the company they work for, more attached to it, and more willing to accept the idea that they are a part of it (Duffy et al., 2012). Employees with strong emotional ties to the company are more likely to put the company’s interests ahead of their own, push themselves and take risks for the good of the company, and to show up to work while ill than employees with weak emotional ties (Brosi & Gerpott, 2022; Duffy et al., 2012; Ferreira et al., 2024). Humayun et al. (2022) assert that vocational calling instils a sense of purpose in employees, motivating them to attend work even when confronted with discomfort or minor personal issues. Simultaneously, when nurses perceive nursing as a “calling”, they develop a profound belief in the irreplaceable value of their contributions to the health and well-being of patients. This conviction is often accompanied by a fear that their absence could disrupt the continuity and quality of patient care (Dirgar et al., 2024). As a result, for nurses, attendance becomes a fundamental means of fulfilling their responsibilities, with this sense of duty acting as a significant positive driver of attendance. Therefore, we propose the following hypothesis:

H1: Career calling is positively correlated to presenteeism.

### The mediation role of workaholism

Workaholism is described as an individual’s overindulgence in work to the extent that they cannot escape it (Schaufeli et al., 2008). It is characterised by putting a great deal of effort into work and even forgoing crucial social, familial, and recreational pursuits (Hadjisolomou et al., 2022). High workaholics constantly think about work, even when not working (Hadjisolomou et al., 2022). According to WCT theory (Duffy et al., 2018), people with a calling are prepared to give up their time and effort in areas other than work to pursue their ideals and the contribution that their employment makes to society. They rationalise abnormal levels of work engagement in order to become obsessed with their jobs, which they view as essential and admirable (Duffy et al., 2018; Ehrhardt & Ensher, 2021). According to this hypothesis, Clinton et al. (2017) discovered that people with a calling were more likely to have dedicated a significant amount of time to their profession, even at the price of personal time off. Also, those who have a high calling are more likely to strive for greater work meaning and prosocial altruism, give of themselves willingly to others at work, set high standards for themselves, take on more responsibilities at work, and experience greater job satisfaction (Duffy & Dik, 2013). Some may even justify unhealthily high levels of work dedication as necessary or commendable given the social or personal values they aspire to, giving them a sense of moral acceptance (Andel et al., 2021). Workaholism is more acceptable among employees, who also enjoy and feel satisfied by it. Consequently, we suggest the following:

H2: Career calling is positively correlated to workaholism.

The link between workaholism and presenteeism will be positive. First, compared to other employees, workaholics frequently have an unquenchable firm urge to work harder than the organisations’ standard demands (Brosi & Gerpott, 2022). They find it challenging to draw a line between work and life because of their lengthy job commitments (Johns, 2010). Individuals exhibiting higher levels of workaholism often struggle to control their impulses to work and report an increased need for the intense pleasure and sense of accomplishment derived from work-related activities, and this passion for their work leads them to remain at the workplace for extended hours and to actively engage in their tasks (Bonebright et al., 2000; Johns, 2010). Consequently, they typically demonstrate high attendance rates and are rarely absent due to minor circumstances. Additionally, nursing-related research indicates that the work environment and professional values of nurses play a crucial role in motivating them to adopt a patient-centred approach and to demonstrate a high level of responsibility (Dirgar et al., 2024). Furthermore, workaholism nurses are particularly influenced by these values; even when they experience physical fatigue or emotional distress, they persist in attending work to ensure that patients receive uninterrupted care (Gillet et al., 2021). Consequently, we put up the following hypothesis:

H3: Workaholism is positively correlated to presenteeism .

H4: Workaholism mediates the relationship between career calling and presenteeism.

### The moderated role of self-compassion

In its broadest meaning, self-compassion refers to compassion for oneself when faced with challenging, unpleasant, or unsuccessful circumstances (Dodson & Heng, 2022; Neff, 2003). Three things make up self-compassion: self-kindness, the sense of common humanity, and mindfulness. The inclination to take care of oneself and comprehend oneself instead of severely criticising and blaming oneself is self-kindness. Realising that no one is flawless and that everyone has the potential to fail, make errors, or engage in harmful habits is a crucial component of having a sense of common humanity. Mindfulness implies paying attention to the here and now with clarity and balance, without denying or clinging to the wrong parts of oneself or life (Dodson & Heng, 2022; Kreemers et al., 2018; Neff, 2003).

Self-compassion can assist people in reducing the negative behavioural impacts of workaholism. Firstly, self-compassion gives you the mental room to reduce unhealthy work states since it is a balanced state of consciousness (Kreemers et al., 2018). Self-compassion entails some transcendence of oneself, a more expansive view of one’s reality, and thus a more realistic and unbiased assessment of one’s experience (Dodson & Heng, 2022; Kreemers et al., 2018; Neff, 2003). According to Schaufeli et al. (2008) and Tóth-Király et al. (2021), workaholism is frequently an excessive indulgence in or rumination on one’s work that causes the person to become demanding and push oneself. Self-compassion enables people to put enough space between themselves and this experience to reduce the excessive demands they place on themselves and to promote self-awareness and comfort (Lanaj et al., 2022).

Second, self-compassion increases a person’s positive psychological strength and serves as a shield against unfavourable circumstances (Lanaj et al., 2022). Happy and optimistic people tend to be more self-compassionate, two crucial elements of mental health (Lanaj et al., 2022). Self-compassion predicts a more significant percentage of happiness than social support (Neff, 2023). Moreover, self-compassion is linked favourably to independent thinking, good emotions, inquiry, curiosity, and reflective and emotional intelligence (Dodson & Heng, 2022; Kreemers et al., 2018; Neff, 2003). More significantly, self-compassion is also linked to feelings of autonomy, competence, and connectedness (Neff, 2003), indicating that it aids in meeting one’s basic needs, which Deci and Ryan (2000) claim are essential to human well-being. As was already said, the ability to accept unpleasant emotions in non-judgemental awareness instead of repressing or rejecting negative parts of one’s self-experience is a sort of self-compassion (Dodson & Heng, 2022; Kreemers et al., 2018; Neff, 2003). So, these psychological benefits of self-compassion can aid people in managing their workaholic tendencies and thereby lessen presenteeism practices. In light of this, we suggest the following hypothesis:

H5: Self-compassion moderates the relationship between workaholism and presenteeism. The positive correlation between workaholism and presenteeism is weaken when self-compassion is high compared to low.

The WCT theory (Duffy et al., 2018) states that workaholism is a mediator between calling and presenteeism. The link between workaholism and presenteeism is then moderated by self-compassion. While working excessively, the person with stronger self-compassion will show less presenteeism. We further hypothesised that self-compassion might influence calling and presenteeism through workaholism; when self-compassion is high, the relationship between calling and presenteeism becomes weaker. Accordingly, in conjunction with H4 to H5, we formulate the hypothesis:

H6: The positive indirect effect of career calling and presenteeism via workaholism will weaken when the individual’s self-compassion is higher.

## Methods

### Participants and procedure

Using the authors’ social networks, we contacted the nursing and human resource management departments of three hospitals in northern China. We used an open recruiting procedure to find full-time nursing staff for the questionnaire. To minimise the impact of common method bias on the relationships among variables, this study utilised the approach proposed by Boekhorst and Halinski (2023) by distributing and collecting online questionnaires from nursing staff across three distinct time periods. At time 1, the nursing staff gave their demographic data and assessed their calling and self-compassion. Two weeks later, the nursing staff was evaluated for their workaholism (Time 2). After two additional weeks, the nursing staff rated their presenteeism (Time 3).

It is also important to note that the data were collected using anonymous responses to minimise personal bias among the respondents. Additionally, variable names were not labelled in the questionnaire to obscure the study’s purpose and the meaning of the questions, thereby ensuring that respondents answered based on their true feelings. The questionnaire was coded for matching purposes, and participants were required to provide the last four digits of their cell phone numbers at each stage. Subjects who completed all three stages of data collection received a payment of 50 RMB from the researcher. All participants provided informed consent prior to the commencement of the study and were free to withdraw at any time during the data collection process without facing any consequences or penalties. The researchers were committed to maintaining the privacy of all procedures and results.

Two hundred fifty-two nursing staff members volunteered to participate, and 218 sets of data were matched, giving us an overall response rate of 86.51%. The final sample included 61.01% married people, 80.73% with bachelor’s degrees or above, and 93.12% women. The average age was 30.93 years (SD = 6.81), and the average number of years worked as a nurse was 8.42 (SD = 7.37). Junior nurses made up 25.68% of the position, junior nurse practitioners 30.28%, nurse practitioners-in-charge 38.53%, and associate chief nurse practitioners 5.50%.

### Measures

For the Chinese translation of the English scale, we used Brislin’s (1980) translation back translation technique approach. Initially, a bilingual person (a management PhD candidate) translated the English scale into Mandarin. A second multilingual applicant for a doctorate in management then reverse-translated the Chinese scale into English. An associate professor of management who is a third party compared the variations between the back-translated and original versions. The three produced the final form after some minor differences were considered. Finally, all surveys were answered in Mandarin. A 5-point Likert scale with a response range of 1 (strongly disagree) to 5 (strongly agree) is available for each measure.

### Career calling

We used the 4-item brief calling scales developed by Dik et al. (2012). An example item is “I have a good understanding of my calling as it applies to my career”. Cronbach’s α was .91.

### Workaholism

We utilised the Chinese version of the Multidimensional Workaholism Scale, originally developed by Clark et al. (2020) and subsequently translated by Xu and Li (2021). This scale comprises four dimensions: motivational, cognitive, affective, and behavioural, with four items dedicated to each dimension. For example, “I always have an inner pressure inside me that drives me to work”. Cronbach’s α was .88.

### Presenteeism

We used the 2-item presenteeism scale developed by Lu et al. (2013). We asked participants about their experiences over six months. The scale includes two items, “Although you feel sick, you still force yourself to go to work” and “Although you have physical symptoms such as headache or backache, you still force yourself to go to work”. Cronbach’s α was .85.

### Self-compassion

We measured self-compassion using five items from Schabram and Heng (2022). An example is, “I try to be understanding and patient towards those aspects of my personality I do not like”. Cronbach’s α was .90.

### Control variables

Referring to previous studies, this paper incorporates gender, age, marital status, education level, tenure, and job title as control variables to minimise the influence of these factors on the findings (Gustafsson Sendén et al., 2016; Van Waeyenberg, 2024). The final results indicated that the analyses conducted with and without control variables yielded nearly equivalent outcomes.

### Analysis strategy

For data analysis and hypothesis testing, we utilised SPSS 23.0 and Mplus 8.0. In order to establish the discriminant validity, we first utilised confirmatory factor analysis. Second, the mediating hypothesis was validated using SPSS macro Process 3.0. In order to generate 95% bias-corrected confidence intervals, we conducted 5000 bootstrap iterations (CIs). Lastly, we tested the validity of the interaction terms using the method proposed by Aiken and West (1991) and tested the moderating hypothesis using a simple slope analysis. Following Preacher et al. (2007), we estimated the indirect impact and the moderated mediation coefficients at high (above one standard deviation) and low (below one standard deviation) values of the moderating variable for the moderated mediation hypothesis.

## Results

### Confirmatory factor analysis

Prior to hypothesis testing, we used Mplus 8.0 to do a series of confirmatory factor analyses to check the distinctness of our variables. The hypothesised 4-factor model – career calling, workaholism, presenteeism, and self-compassion – was contrasted with other alternative models. The findings showed that the hypothesised model performed better than numerous competing models (*χ*^*2*^(480) = 1079.73, CFI = .95, TLI = .94, RMSEA = .06, SRMR = .04) in terms of how well it suited the data (see Table 1).

**Table 1.** Confirmatory factor analysis.

Model	χ^2^ (*df*)	χ^2^ */df*	Δχ^2^	RMSEA	CFI	TLI	SRMR
4-factor model^a^	172.06(81)	2.12		.07	.95	.94	.06
3-factor model^b^	292.44(84)	3.48	120.38***	.11	.89	.87	.08
3-factor model^c^	327.19(84)	3.90	155.13***	.12	.88	.85	.11
3-factor model^d^	341.42(84)	4.06	169.36***	.12	.87	.84	.09
2-factor model^e^	765.78(86)	8.90	593.72***	.19	.65	.58	.17
Single-factor model^f^	926.33(87)	10.65	754.27***	.21	.57	.49	.18

*N* = 218; a. Hypothesized 4-factor model; b. Combining career calling and presenteeism into one factor; c. Combining workaholism and presenteeism into one factor; d. Combining career calling and presenteeism into one factor; e. Combining career calling, workaholism, and presenteeism into one factor; f. All factors were combined into a single factor.

***p < 0.001, **p < 0.01, *p  < 0.05.

### Descriptive statistics

Table 2 shows means, standard deviations, and correlations for variables. According to Table 2, career calling is positively correlated with presenteeism (*r* = .28, *p* < .001), workaholism (*r* = .41, *p* < .001), and self-compassion (*r* = .21, *p* = .002); workaholism is positively correlated with presenteeism (*r* = .26, *p* < .001).

**Table 2.** Means, standard deviations, correlations, and reliabilities.

	M	SD	1	2	3	4	5	6	7	8	9	10
1 Gender	1.93	.25	–									
2 Age	30.93	6.81	.10	–								
3 Educational Level	2.87	.51	−.07	.33***	–							
4 Marital Status	1.39	.49	−.12	−.69***	−.24***	–						
5 Tenure	8.42	7.37	.20**	.95***	.21**	−.63***	–					
6 Title	2.24	.90	.09	.80***	.41***	−.64***	.73***	–				
7 Career Calling	3.98	.61	.17*	.18**	.07	−.13	.20**	.15*	(.91)			
8 Presenteeism	3.69	.67	.23**	.26***	.09	−.26***	.29***	.23**	.28***	(.88)		
9 Workaholism	3.18	.57	.05	.12	.15*	−.10	.12	.14*	.41***	.26***	(.85)	
10 Self-compassion	3.51	.72	.03	−.03	−.01	.13*	−.03	.01	.21**	.02	.12	(.90)

*N* = 218; ***p < 0.001, **p < 0.01, *p < 0.05; Cronbach’s alpha is in parentheses along the diagonal.

### Hypothesis tests

Table 3 shows the path analysis results. Hypothesis 1 and 2 posited that career calling positive is related to workaholism and presenteeism. As shown in model 1, there was a significant positive relationship between career calling and workaholism (*b* = .37, *p* < .001). There also was a significant positive relationship between workaholism and presenteeism (model 2; *b* = .19, *p* = .02). The results support Hypotheses 1 and 2.

**Table 3.** Path analysis results.

	Workaholism	Presenteeism	Presenteeism
	Model 1	Model 2	Model 3
	B(SE)	B(SE)	B(SE)
Constant	1.72(.69)*	2.15(.82)**	2.72(.81)*
Gender	−.06(.15)	.38(.18)*	.36(.18)*
Age	−.01(.02)	−.02(.02)	−.01(.02)
Educational	.13(.08)	.04(.10)	.04(.09)
Marital Status	−.03(.10)	−.19(.12)	−.20(.12)
Tenure	.01(.02)	.02(.02)	.01(.02)
Title	.04(.07)	−.002(.08)	.01(.08)
Career Calling	.37(.06)***	.16(.08)*	.12(.08)
Workaholism		.19(.08)*	.16(.08)*
Self-compassion			.01(.06)
Workaholism * Self-compassion			−.22(.07)***
R^2^	.18	.19	.23
F	6.64***	6.08***	6.03***

*N*  = 218; ***p < 0.001, **p < 0.01, *p  < 0.05; Unstandardized coefficients are reported. Standard errors are in parentheses.

We tested the mediation hypothesis using the Model 4 in SPSS macro process 3.3. The results indicated a significant positive relationship between career calling and presenteeism (model 2; *b* = .16, *p* = .04). The mediation effect was further assessed using the Bootstrap method, with a total of 5000 bootstrap samples. The results showed a significant positive indirect effect of career calling on presenteeism via workaholism (estimate = .07, 95% bias-corrected CI = [.004, .14], excluding 0). Therefore, Hypothesis 3 was supported.

**Figure 2.**
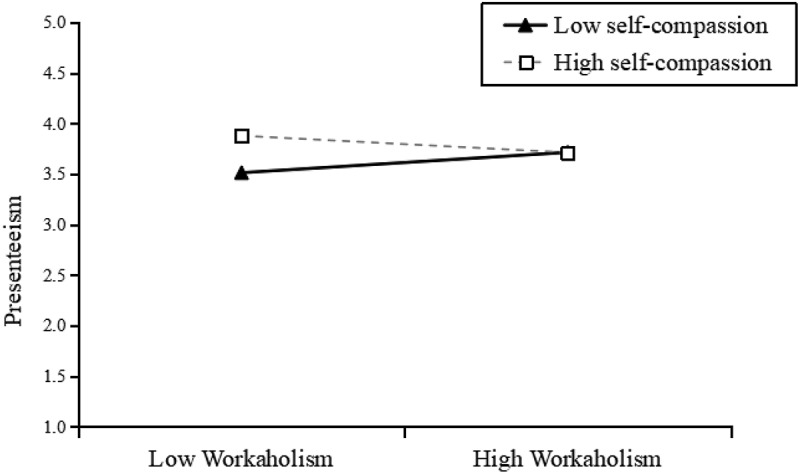
The interactive effect of workaholism and self-compassion on Presenteeism.

Hypothesis 4 posited that self-compassion moderates the relationship between workaholism and presenteeism. We tested the moderation hypothesis using the Model 14 in SPSS macro process 3.3. As shown in model 3, there was a significant negative interactive effect of workaholism and self-compassion on presenteeism (*b* = −.22, *p* = .002). We then conducted the simple slope test (Figure 2) at different values of the moderator (±1 SD of mean) developed by Aiken and West (1991). The positive relationship between workaholism and presenteeism is significantly weaker for employees with high self-compassion than those with low self-compassion (*b* = −.001, *p* = .99, *n.s*., +1 SD; *b* = .32, *p* < .001, −1 SD). The results support Hypothesis 4.

Finally, to test Hypothesis 5, we employed 5,000 Bootstrap samples and calculated bias-corrected 95% confidence intervals to assess the mediating effect of workaholism on the relationship between career calling and presenteeism at varying levels of self-compassion (both high and low). The results indicated that career calling had a significant negative indirect effect on presenteeism via workaholism under the low self-compassion (estimate = .12, SE = .04, 95% bias-corrected CI = [.04, .20]), while had a nonsignificant indirect effect under the high self-compassion (estimate = .001, SE = .04, 95% bias-corrected CI = [−.07, .08]). Moreover, the indirect effects were significantly different between the two conditions (estimate = −.11, SE = .04, 95% bias-corrected CI = [−.20, −.03]).

## Discussion

We investigated the negative effects of relying on nurse presenteeism and the protective features of self-compassion based on the WCT theory (Duffy et al., 2018) and compassion literature (Neff, 2003, 2023). In particular, we discovered that self-compassion reduced the association between career calling and presenteeism via workaholism. We also determined that workaholism plays a mediation role between career calling and presenteeism.

### Theoretical implications

This paper presents several theoretical implications. First, we find that presenteeism and workaholism among nurses are positively correlated with career calling. While these results may benefit the organisation, they are detrimental to the physical and emotional health of the workers (Bonebright et al., 2000; Hadjisolomou et al., 2022). This finding enhances the application of WCT theory within the context of healthcare nursing, elucidating how an individual’s perception of their career calling translates into behavioural performance (presenteeism) among nurses. This contribution deepens the understanding of the mechanisms through which the abstract concept of career calling operates in nurses’ daily work practices. WCT theory emphasises the individual’s perception of meaning and intrinsic motivation for work, and this study clarifies the pathways through which this intrinsic motivation positively influences nurses’ attendance behaviours.

Second, the identification of workaholism as a mediating variable enhances the WCT theory. This discovery suggests that career calling does not directly drive presenteeism; rather, it exerts an indirect influence by fostering a workaholic-like state of work engagement. Such a state is characterised by nurses’ elevated levels of enthusiasm and total commitment to their work, alongside an excessive preoccupation with work-related matters. Workaholism thus acts as an intermediary between the psychology of career calling and attendance behaviour. Recognising this mediating effect provides a more nuanced understanding of the behaviour-driven processes within WCT, illuminating the intricate psychological and behavioural transmission mechanisms that may exist and completing the causal chain of the theory. Moreover, this finding is consistent with prior research indicating that presenteeism is triggered by either career calling or workaholism (Hadjisolomou et al., 2022; Hirschi et al., 2019).

Finally, the existing literature remains unclear regarding the boundary conditions of career calling in influencing nurse presenteeism. We draw our inspiration from the research on self-compassion (Neff, 2003, 2023), which contends that people can actively manage unpleasant experiences and feelings. Our study investigates the moderating role of nurse self-compassion in the relationship between nurse workaholism and presenteeism, thereby expanding the boundaries of WCT theory. The findings suggest that an individual’s affective factors, such as self-compassion, can serve as either a buffer or enhancement in the behavioural chain triggered by career calling, rendering the theory more dynamic and contextually adaptable. This research contributes to a deeper understanding of how career calling differentially influences work behaviours based on individual traits, offering new insights into WCT theory by considering the dimensions of individual affective traits. Furthermore, Like in prior research, we discovered that having self-compassion helps people deal with negative self-states and mitigates their negative impacts. Self-compassion is the ability to care for and understand oneself (Leary et al., 2007; Schabram & Heng, 2022). When faced with failure or setbacks, self-compassion promotes people to adopt more emotion-centred coping mechanisms, including acceptance, constructive reframing, and development, reducing the onset of negative emotions (Chwyl et al., 2021; Leary et al., 2007).

### Practical implications

Our study has salient practical implications. First, the possible negative impacts of career calling on a person’s state and conduct are shown by our research. These findings prompt hospital administrators to believe that a career calling increases work commitment. Nevertheless, if there is a significant focus on career calling, nurses may be compelled to work longer hours out of a sense of duty, and working too many hours may prevent them from recovering fully (Andel et al., 2021). Long-term effects might be detrimental to an employee’s personal life, physical and mental health, and work performance. In order to prevent possible harm from professional work from affecting nurses’ personal lives, physical health, and mental health, hospital managers need to pay attention to work-life balance, increase support for welfare programs, and care for staff. We can only encourage human resources’ healthy and sustainable development by consistently enhancing the conditions for nurses’ professional and personal lives.

Second, managers should address workaholism as a severe psychological condition. Organisations should take necessary action to deal with those already showing signs of workaholism. They can help such groups recognise and lessen the harm and suffering caused by such workaholism by communicating with managers or seeking professional counselling. They can also help such groups understand the harm that workaholism brings to themselves and their families and to interpersonal and organisational relationships. Also, workaholics might benefit from psychological treatment to improve their single-work addiction behaviour on a cognitive level, enhance their interpersonal relationships, and make the most of their free time.

Finally, Individuals and organisations should be aware of self-compassion’s crucial role in overcoming suffering. Self-compassion may be taught; individuals can become more understanding of themselves (Neff, 2023). According to specific research, even when participants are told to view their difficulties with self-compassion, they can still have beneficial results (Rahimi-Ardabili et al., 2018). Organisations may think about compassion training to create a supportive work environment and guarantee that the emotional health of organisational members is satisfied.

### Limitations and future directions

The study has several potential limitations as well. First, we employed a time-lagged approach to better distinguish the variables’ correlations, allowing respondents to avoid the burden of recalling all pertinent information simultaneously. This approach enhances the accuracy of the data at each stage, reflecting the circumstances more precisely at that specific time. Additionally, collecting data in stages mitigates the bootstrapping influence of earlier variables on subsequent ones, thereby increasing the independence of the data. However, this is still a cross-sectional design and causality cannot be inferred from the data. There may have been daily fluctuations in the primary variables discussed in the article. Future research could explore the use of an experience sampling method, which involves collecting immediate responses from individuals at multiple points in time. This approach would allow for an assessment of how a nurse’s career calling influences presenteeism through workaholism, and subsequently, presenteeism itself. Such a methodology would facilitate the acquisition of more realistic dynamic causal data from individuals.

Second, the sample was overwhelmingly made up of women since we concentrated on nurses. We encourage future research to consider expanding the findings of this study to male caregivers as more males enter the nursing profession. Also, although several primary study variables in the article showed statistically significant results, they exhibited relatively weak trends. Future research could broaden the scope to encompass a wider range of occupations, including those beyond the nursing field, such as those held by doctors, teachers, and other professionals, to further evaluate the reliability of the findings.

Finally, even though we looked into the negative aspects of the career calling, we just looked at nurses’ attitudes and behaviour. We did not assess nurses’ psychological, physical, or occupational performance. These results are crucial and directly affect the person. A key area of research to examine the results of calling would be family outcomes. Consequently, future studies might supplement calling outcomes by examining these individual and family results.

Furthermore, because our study was done in China and all participants were Chinese, we could not examine how calling varied among cultures. As a result of the increased focus on collectivism and devotion in China, presenteeism and career callings are more likely to develop there (Kitayama & Markus, 2000; Li et al., 2022; Sullivan et al., 2012). However, western cultures may value individuality more, leading to lower calling and presenteeism percentages. As a result, we expect that subsequent researchers will be able to reproduce and expand upon the findings of this study in different cultures.

## Conclusions

Considering that calling may have a negative side, it is essential to comprehend how it happens and the safeguards that lessen this negative impact. Based on a WCT theory (Duffy et al., 2018) and the compassion view (Neff, 2003, 2023), we found that individuals with high self-compassion generate less presenteeism when confronted with calling through workaholism. Our work serves as a starting point for future research examining the negative aspects of career calling and the safeguards that guard against them.

## Data Availability

The data that support the findings of this study are available on request from the corresponding author. The data are not publicly available due to privacy or ethical considerations.

## Note

1. In the context of the pandemic, the dedication of healthcare workers has been highlighted by leaders worldwide, including WHO Director-General Tedros Adhanom Ghebreyesus, who referenced the exemplary service of Ms. Lopez, a nurse, in his opening remarks at the media briefing on COVID-19 on 17 July 2020. https://www.who.int/director-general/speeches/detail/who-director-general-s-opening-remarks-at-the-media-briefing-on-covid-19—17-july-2020.
